# Testicular germ cell tumours’ clinical stage I: comparison of surveillance with adjuvant treatment strategies regarding recurrence rates and overall survival—a systematic review

**DOI:** 10.1007/s00345-022-04145-6

**Published:** 2022-09-15

**Authors:** Christian G. Ruf, Stefanie Schmidt, Sabine Kliesch, Christoph Oing, David Pfister, Jonas Busch, Julia Heinzelbecker, Christian Winter, Friedemann Zengerling, Peter Albers, Karin Oechsle, Susanne Krege, Julia Lackner, Klaus-Peter Dieckmann

**Affiliations:** 1 Department of Urology, Bundeswehrkrankenhaus (German Federal Armed Forces Hospital), Ulm, Germany; 2 UroEvidence@Deutsche Gesellschaft Für Urologie, Berlin, Germany; 3 Centre of Reproductive Medicine and Andrology, Department of Clinical and Surgical Andrology, University Hospital Münster, Münster, Germany; 4 II. Medical Clinic and Polyclinic, University Hospital Hamburg-Eppendorf, Hamburg, Germany; 5 Department of Urology, University Hospital Cologne, Cologne, Germany; 6 Department of Urology, Vivantes Clinics Am Urban, Berlin, Germany; 7 Department of Urology and Paediatric Urology, Faculty of Medicine, Saarland University Medical Centre and Saarland University, Homburg, Saar, Germany; 8 Urologie Neandertal (Regional Joint Practice), Erkrath, Germany; 9 Department of Urology, University Hospital Ulm, Ulm, Germany; 10 Department of Urology, University Hospital Düsseldorf, Düsseldorf, Germany; 11 Clinic for Urology, Pediatric Urology and Urological Oncology, KEM, Protestant Hospital Essen-Mitte, Essen, Germany; 12 Department of Urology, Asklepios Klinik Altona, Hamburg, Germany

**Keywords:** Germ cell tumour, Clinical stage I, Active surveillance, Radiotherapy, Chemotherapy, Overall survival, Recurrence rate, Systematic review

## Abstract

**Purpose:**

Testicular germ cell tumours (GCTs) represent the most common malignancy in young adult males with two thirds of all cases presenting with clinical stage I (CSI). Active surveillance is the management modality mostly favoured by current guidelines. This systematic review assesses the treatment results in CSI patients concerning recurrence rate and overall survival in non-seminoma (NS) and pure seminoma (SE) resulting from surveillance in comparison to adjuvant strategies.

**Methods/systematic review:**

We performed a systematic literature review confining the search to most recent studies published 2010–2021 that reported direct comparisons of surveillance to adjuvant management. We searched Medline and the Cochrane Library with additional hand-searching of reference lists to identify relevant studies. Data extraction and quality assessment of included studies were performed with stratification for histology (NS vs. SE) and treatment modalities. The results were tabulated and evaluated with descriptive statistical methods.

**Results:**

Thirty-four studies met the inclusion criteria. In NS patients relapse rates were 12 to 37%, 0 to 10%, and 0 to 11.8% for surveillance, chemotherapy and for retroperitoneal lymph node dissection (RPLND) while overall survival rates were 90.7−100%, 91.7−100%, and 97−99.1%, respectively. In SE CSI, relapse rates were 0−22.3%, 0−5%, and 0−12.5% for surveillance, radiotherapy, chemotherapy, while overall survival rates were 84.1−98.7%, 83.5−100%, and 92.3−100%, respectively.

**Conclusion:**

In both histologic subgroups, active surveillance offers almost identical overall survival as adjuvant management strategies, however, at the expense of higher relapse rates. Each of the management strategies in CSI GCT patients have specific merits and shared-decision-making is advised to tailor treatment.

**Supplementary Information:**

The online version contains supplementary material available at 10.1007/s00345-022-04145-6.

## Introduction

Clinical stage I (CSI) is the most common stage at primary diagnosis of testicular germ cell tumours (GCTs) involving 50% of patients with non-seminoma (NS) and as many as 80% of those with pure seminoma (SE) [1, 2].

CSI is characterized by the absence of metastases upon clinical, radiological and biochemical examinations. However, microscopic metastatic seeds may escape detection, because imaging techniques involve a failure rate of 20–30% and serum tumour marker expression is present in only 50–60% of non-seminomas and in no more than 30% of seminomas.

Traditionally, clinical management of CSI patients involved adjuvant treatment in all patients to prevent progression to overt metastatic disease. In seminoma, abdominal radiotherapy used to be the standard of care until adjuvant carboplatin therapy proved to be non-inferior [3]. In non-seminoma, retroperitoneal lymph node dissection (RPLND) used to be the standard of care until the late 1990ies [4].

The rationale for surveillance strategies in CSI patients is principally based on Hippocrates` rule of “primum nil nocere” and it specifically rests on the experience that the majority of these patients are already cured with orchiectomy alone. Thus, adjuvant therapy for the entire group of CSI patients would involve overtreatment with the risk of unnecessary treatment-related toxicity in a substantial number of CSI patients. The concerns regarding such toxicity involve mainly cardiovascular diseases and secondary malignant neoplasms in patients undergoing radiotherapy. Further of concern are surgical complications and particularly loss of ejaculation in patients undergoing RPLND, while patients receiving adjuvant cisplatin-based chemotherapy might be burdened by a large spectrum of metabolic, neurological and endocrinological toxicities including impairment of fertility and secondary malignancies. Justification for the use of surveillance instead of adjuvant treatment emerged from the excellent cure rates of relapsing patients achieved with chemotherapy and additional surgery in select cases. Support for the concept of surveillance comes from early prospective studies that revealed virtually equivalent overall survival rates with both surveillance and adjuvant therapy. However, it was also shown in these studies that the population of CSI patients is quite heterogeneous with regard to the risk of progression. There are some subgroups involving high probabilities of progression that are characterized by histologic type (seminoma and non-seminoma) and also by histopathological features like lymphovascular invasion (LV1) in non-seminoma and primary tumour size > 4 cm in seminoma patients [5]. Other factors such as percentage of embryonal carcinoma or presence of teratoma in non-seminoma patients and rete testis invasion in seminoma patients did not reach international consensus so far [6]. With the knowledge of specific risk factors for progression, the option of risk-adapted management of CSI patients evolved with adjuvant treatment in cases with risk factors and surveillance in those without. Currently, most of the international guidelines generally recommend surveillance for CSI patients but offer the options of adjuvant therapy in high-risk cases. Thus, the management of GCT patients with CSI must be based on a number of individual factors including personal preferences of the patient [7]. The aim of the present review is to summarize the results of the most recent studies regarding relapse rates and overall survival in various subgroups of CSI patients and to thereby provide caregivers of GCT patients a sound basis for individual patient consultation and joint treatment decision-making.

## Methods

The present analysis is based on a systematic literature search that was conducted for the elaboration of the first German clinical practice guideline [8]. Here, we present the updated results on patients with GCT at CSI.

## Systematic literature search

We performed a systematic review of the most recent literature (January 2010 to February 2021) by searching the biomedical databases Medline (via Ovid) and the Cochrane Library. We only considered randomized clinical trials and observational comparative studies focusing on patients with CSI GCT (seminoma and non-seminoma) receiving surveillance and/or adjuvant management modalities such as radiotherapy, chemotherapy, or retroperitoneal lymph node dissection (RPLND). The search was limited to full text publications and to articles in English or German language. An additional search for unpublished data and ongoing studies was conducted in clinical trial registers (clinicaltrial.gov/ and www.who.int/ictrp/). In cases of missing information for studies identified in the trial registries an enquiry to the study coordinators was conducted. Additionally, hand-searches of the reference lists of included studies were performed to disclose additional, relevant studies. Single arm studies were excluded in systematic literature search but considered in the discussion. Two studies encompassing ≤ 50 patients were excluded to reduce the risk of bias. Details of the search strategies are given in the appendix. Our study endpoints were recurrence rates and overall survival stratified for the subgroups of SE and NS, respectively.

## Literature screening, data extraction and quality assessment

Relevant data of the studies were documented in evidence tables and analyzed with descriptive statistical methods. The study quality was appraised by one author (JL) using the Cochrane risk of bias tool (Cochrane Handbook) for randomized studies and the SIGN checklist for cohort studies. The level of evidence was rated according to the Oxford criteria [9]. In any case of uncertainty, a second author (CGR or SS) was involved to reach consensus by discussion.

## Non-seminoma

Following orchiectomy, there are basically two therapeutic options for non-seminoma patients in CSI: surveillance or one or two cycles of chemotherapy with cisplatin, etoposide and bleomycin (BEP). In selected cases, RPLND can be another option.

The most important prognostic marker for the presence of occult metastases is lymphatic vascular invasion (LVI), defined as direct tumour spread into lymphatic and/or blood vessels [6]. We stratified the studies according to the treatment modality and pathohistological findings and compared relapse and overall survival rates across the stratification arms.

## Seminoma

Risk stratification for relapse in seminoma is mostly based on tumour size > 4 cm, although this parameter has also been considered as a continuous variable to predict the risk of relapse [5, 10]. We tabulated the studies identified in the search according to the treatment modality (surveillance, carboplatin chemotherapy, or radiotherapy) and to tumour size and compared the relapse rates and the overall survival rates across the stratification arms.

## Results

### Summary of the evidence table–general results

**Fig. 1:**
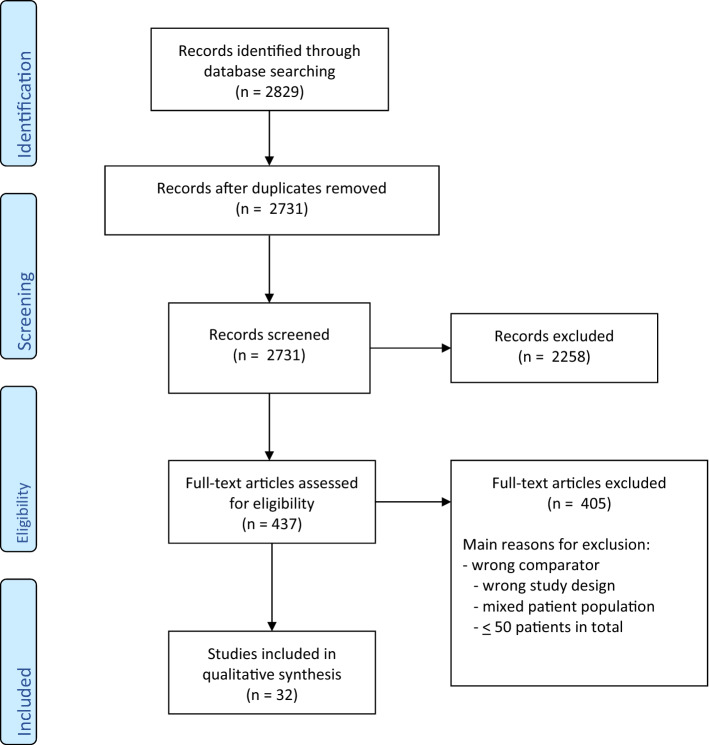
Study flow diagram

A total of 32 studies met the inclusion criteria. The PRISMA search process is shown in Fig. 1.

Nineteen studies related to seminoma, 10 to non-seminoma, and 3 to mixed GCT populations. Eight studies had been conducted prospectively thereof seven cohort studies and one randomized controlled trial (RCT). Twelve studies assessed adjuvant chemotherapy, four radiotherapy, 12 studies evaluated both chemotherapy and radiotherapy, 4 summarized various different adjuvant treatment methods. The additional hand-search revealed six further studies, two reported results for seminoma, one for non-seminoma and three for both histologic subgroups. As these reports represented mostly single-arm studies, they had to be excluded for the systematic literature search according to our exclusion criteria of a systematic review. Yet, the results of these studies are presented in separate tables and are also included in the discussion.

### Non-seminoma–relapse rate

Table 1 lists the results on surveillance in NS CSI reported from seven comparative studies encompassing a total of almost 950 patients. Without employing risk factors, the relapse rates range from 12 [11] to 25% [12]. In cases with lymphovascular invasion (LVI) surveillance resulted in a progression rate of 60% (high risk), while the rate is 13 to 17.3% for LVI negative tumours (low risk).

**Table 1:** Relapse rates (%) in non-seminoma CSI patients managed by surveillance (with and without risk stratification)

First author	Year	Design	Study period	*n*	All NS CSI	High risk	Low risk
Dieckmann [13]	2018	Retrospective cohort study	2008–2017	9	22%		
Shinoda [11]	2018	Retrospective cohort study	2005–2008	132	12%		
Ondrusova [14]	2017	Retrospective cohort study	1992–2017	301			17.3%
Ondrus [15]	2015	Prospective cohort study	1992–2014	287			16.7%
Kobayashi [12]	2013	Retrospective cohort study	1980–2008	36	25%		
Lv [16]	2013	Retrospective cohort study	1997–2011	37	22%		
Tandstad [17]	2010	Randomised controlled trial	1995−1998	5 high risk 124 low risk		60%	13%

Information about LVI was provided in three studies only. Table 2 lists data from the four single-arm retrospective case series on active surveillance with up to 1226 patients included. Relapse rate ranges from 19 to 37% in all NS and up to 42.4% in high-risk patients.

**Table 2:** Relapse rates (%) in non-seminoma CSI patients managed by surveillance (with and without risk stratification), non-comparative studies

First author	Year	Design	Study period	*n*	All NS CSI	High risk	Low risk
Daugaard [18]	2014	Retrospective case series	1984–2007	1226	30.6%*		
Kollmannsberger [19]	2015	Retrospective case series	1998–2010	1139	19%**	44%	14%
Lago-Hernandez [20]	2015	Retrospective case series	1997–2013	135	37%	78%	25%
Nayan [21]	2017	Retrospective case series	1980–2014	464	27.2%	42.4%	17.3%

^*^this study did not provide separate relapse rates in high-risk patients but reported a hazard ratio for relapse of HR = 1.57 for the presence of LVI (*p* < 0.01)

^**^Only 16% of the entire NS population were high-risk cases (LVI +)

Relapse rate for CSI NS patients undergoing adjuvant chemotherapy with two cycles of BEP or CVB ranged between 0 [12–14] and 5.6% [15]. Tandstad [16] reported data for 40 low-risk non-seminoma in 2010, undergoing chemotherapy with a non-standard regimen (vinblastine instead of etoposide), of whom four (10%) patients relapsed. All other chemotherapy studies included high-risk patients only.

Relapse rates following RPLND were reported in two comparative studies only, comprising a total number of 12 and 34 patients, and ranged from 0 [14] to 11.8% [15].

### Non-seminoma–overall survival

Overall survival (OS) was uniformly > 90% with only slight differences between surveillance and other treatment modalities implying that most of the relapses can be cured with appropriate treatment. Minor differences of OS rates also relate to 5 years’ observation intervals opposed to 10 years’ periods. Details are listed in Table 3. Data from the two single-arm retrospective case series are shown in Table 4.

**Table 3:** Overall survival (%) in non-seminoma patients CSI stratified by treatment modality

Reference	Year	Design	Study period	*n*	Surveillance	*n*	Chemotherapy	*n*	RPLND
Kamran [23]	2018	Retrospective Cohort study	2004–2012	411	95%^#^				
Ondrusova [14]	2017	Retrospective	1992–2017	301	98%	184	99.5%		
		Cohort study							
Weiner [24]	2017	Retrospective analysis of National cancer database	2004–2013		Clinical stage IA		Clinical stage IA		Clinical stage IA
				2873 (IA)	97.3%^#^	531 (IA)	98.0%^#^	676 (IA)	99.1%^#^
					94.2%*		95.1%*		97.5%*
					Clinical stage IB		Clinical stage IB		Clinical stage IB
				1195 (IB)	96.5%^#^	882 (IB)	96.0%^#^	503 (IB)	97.8%^#^
					95.8%*		91.7%*		97.0%*
Yap [25]	2017	Retrospective cohort study	1988–2010	1903	97%^#^	962	92%^#^	1049	98%^#^
Ondrus [15]	2015	Prospective cohort study	1992–2014	287	97.9%	167	99.4%		

^*^Ten year overall survival, ^#^5-year OS

**Table 4:** Overall survival (%) in non-seminoma patients CSI undergoing surveillance, non-comparative studies

Reference	Year	Design	Study period	*n*	Surveillance
Daugaard [18]	2014	Retrospective case series	1984–2007	1226	97.6%^#^ 96.2%*
Kollmannsberger [19]	2015	Retrospective case series	1998–2010	1139	99.4% #

*Ten year overall survival, ^#^5-year OS

### Seminoma–general results

A total of 22 cohort studies (5 of them prospective) reported results for patients with seminomas. By comparing to active surveillance, five studies evaluated chemotherapy, ten both chemotherapy and radiotherapy, five radiotherapy, and two summarized different adjuvant treatment modalities. Sample sizes of the studies ranged from 74 to 6700 patients. Five additional single-arm retrospective case studies on active surveillance, including up to 1344 patients, are shown in Table 5.

**Table 5:** Relapse rates in seminoma CSI patients managed by surveillance with and without risk stratification, non-comparative studies

First author	Design	Year	Study period	*n*	All	≥3 cm	< 3 cm
Cummins [33]	2010	Retrospective case series	1984–2007	1226	13%		
Kollmannsberger [34]*	2015	Retrospective case series	1998–2010	1344	13%		
Lago-Hernandez [35]	2015	Retrospective case series	1997–2013	131	15%		
Nayan [36]	2017	Retrospective case series	1980–2014	775		20.3%	12.2%
Mortensen [37]	2014	Retrospective national data base	1984−2008	1954	18.8%		

*Overlap with Swenoteca data (31)

### Relapse rate

Details regarding relapse rates in seminoma patients are listed in Tables 5, 6, 7. There are only five studies reporting relapse rates stratified for subgroups with and without risk factors. With respect to adjuvant chemotherapy, only one study separated one and two cycles of carboplatin treatment and found relapse rates of 5 and 1.5%, respectively [17].

**Table 6:** Relapse rates in seminoma CSI patients managed by surveillance with and without risk stratification

First author	Design	Year	Study period	*n*	All	≥ 4 cm	< 4 cm
Aparicio [18]	Prospective cohort study	2018	2013–2015	71	7%		
Tyrrell [19]	Retrospective cohort study	2018	2004–2016	326	6.1%		
Terbuch [20]	Retrospective cohort study	2017	1994–2013	312	11.2%		
Dieckmann [17]	Prospective cohort study	2016	2008–2013	256	8.2%	7.8%	8.6%
Tandstad [21]	Prospective cohort study	2016	2007–2010	422		15.5%	4.0%
Bilici [22]	Retrospective cohort study	2015	1997–2013	72	22.3%		
Ondrusova [23]	Retrospective cohort study	2015	2008–2015	74			9.5%
Aparicio [24]	Retrospective cohort study	2014	1994−2008	396			14.8%
Haugnes [25]	Retrospective cohort study	2014	1986–2010	75	10.7%		
Kobayashi [12]	Retrospective cohort study	2013	1980–2008	61	6.6%		
Leung [26]	Retrospective cohort study	2013	1981–2004	484	15%		
Khader [27]	Retrospective cohort study	2012	2003–2010	3	0%		
Mahantshetty [28]	Retrospective cohort study	2012	1990–1998	41	11%		
Aparicio [29]	Prospective cohort study	2011	2004–2008	153	11.9%	16.3%	6.5%
Kollmannsberger [30]	Retrospective cohort study	2011	1999–2008	313	19.3%		
Tandstad [31]	Prospective cohort study	2011	2000–2006	512	14.3%		
Kamba [32]	Retrospective cohort study	2010	1985–2006	186	21%

**Table 7:** Relapse rates in seminoma CSI patients managed by radiotherapy or carboplatin chemotherapy

First author	Design	Year	Study period	*n*	Adjuvant chemotherapy	*n*	Adjuvant radiotherapy
Aparicio [18]	Prospective cohort study	2018	2013–2015	64	1.6%		
Terbuch [20]	Retrospective cohort study	2017	1994–2013	37 (1 × Carbo)	8.1%	57	1.8%
Dieckmann [38]	Prospective cohort study	2016	2008–2013	362 (1 × Carbo) 66 (2 × Carbo)	5% (1 × Carbo) 1.5% (2 × Carbo)	41	2.4%
Tandstad [21]	Prospective cohort study	2016	2007–2010	469	2.2% low risk 9.3% high risk		
Bilici [22]	Retrospective cohort study	2015	1997–2013	80	1.2%	130	7.7%
Ondrusova [23]	Retrospective cohort study	2015	2008–2015	16	12.5%		
Aparicio [24]	Retrospective cohort study	2014	1994−2008	348	3.2%		
Haugnes [25]	Retrospective cohort study	2014	1986–2010	106	0%	17	1.9%
Aparicio [29]	Prospective cohort study	2011	2004–2008	74	2%		
Kollmannsberger [30]	Retrospective cohort study	2011	1999–2008	73	2%	159	2%
Tandstad [31]	Prospective cohort study	2011	2000–2006	188	3.9%	481	0.8%
Kamba [32]	Retrospective cohort study	2010	1985–2006	57	6%	182	6%
Diminutto [39]	Retrospective cohort study	2016	2005−2015	115	5.2%		

### Overall survival

Data on overall survival (OS) for CSI SE are summarized in Tables 8 and 9 with stratification for surveillance, carboplatin chemotherapy, and radiotherapy, respectively. Most of the studies report OS rates > 90%, and there is only little variation among the three treatment modalities. However, some variation relates to different observation periods (5-year, 10-year or 20-year OS rates). Cancer-specific survival is usually superior to OS as specified by Jones [18] et al. who noted a 20-year overall survival rate of 83.5% but a cancer-specific survival in as many as 98.2%.

**Table 8:** Overall survival rates in seminoma CSI patients according to treatment modality

Reference	Year	Design	Study period	*n*	Surveillance	*n*	Chemotherapy	*n*	Radiation
Aparicio [18]	2018	Prospective cohort study	2013–2015	S71	100% ^+^	64	100% ^+^		
Kamran [41]	2018	Retrospective cohort study	2004–2012	227	99%^#^	65			
Dieckmann [38]	2016	Prospective cohort study	2008–2013	256	100%			41	100%
Tandstad [21]	2016	Prospective cohort study	2007–2010	422	99.2%^#^ 96.8%*	469	98.9%^#^ 98.5%*		
Bilici [22]	2015	Retrospective cohort study	1997–2013	72	100%^#^	80	92.3%^#^	130	97.4%^#^
Ondrusova [23]	2015	Retrospective cohort study	2008–2015	74	100%	16	100%		
Jones [40]	2013	Retrospective cohort study	1973–2003	1499	95.0%^#^ 92.2% * 84.1%^−^			5265	97.7%^#^ 94.8%* 83.5%^−^
Leung [26]	2013	Retrospective cohort study	1981–2004	484	98.6%^#^ 97.7%*			280	97.2%^#^ 91.4%*
Aparicio [29]	2011	Prospective cohort study	2004–2008	153	100%^+^	74	100%^+^		
Tandstad [31]	2011	Prospective cohort study	2000–2006	512	98.4%^#^	188	99.2%^#^	481	98.7%^#^
Kamba [32]	2010	Retrospective cohort study	1985–2006	186	100%*	57	100%*	182	99.4%*

−20-year, *10-year, ^#^5-year and ^+^3-year overall survival

**Table 9:** Overall survival rates in seminoma CSI patients undergoing surveillance, non-comparative studies

First author	Design	Year	Study period	*n*	All
Cummins [33]	2010	Retrospective case series	1984–2007	1226	96.5%
Kollmannsberger [34]	2015	Retrospective case series	1998–2010	1344	99%
Mortensen [37]	2014	Retrospective population-based study	1984−2008	1954	99.3%*

*Disease-specific 15-year survival

## Discussion

The present study revealed a number of noteworthy results. First, the relapse rate of non-seminoma CSI patients undergoing surveillance without risk stratification is around 30%. Second, patients with lymphovascular invasion will relapse in 40–60% if no adjuvant therapy is applied. Third, adjuvant chemotherapy reduces the relapse rate to < 5%. Fourth, overall survival is > 95% irrespective of post-orchiectomy management modality. Fifth, in seminoma, the relapse rates upon surveillance are somewhat lower than in NS CSI, but here again, survival rates are > 95% irrespective of adjuvant management modality.

With regard to the methodology of the present review, it must be stated that the search algorithm was tailored to exclusively identify comparative studies published in the time-span 2010–2021, aiming to identify first, the most recent studies, and second, the papers of highest methodological quality, i.e. those employing randomized trials or studies that systematically compared surveillance with adjuvant management modalities. Only 34 studies matching these conditions were retrieved by the algorithm. This comparatively low number may relate to the short time-span of eleven years of the search and most probably to the world-wide low incidence of GCTs which precludes meaningful large-scale studies with appropriate observation times to be conducted within this window of time. Furthermore, several high-impact studies published during 2010–2021 were originally not identified by the search because of their non-comparative, single-arm design. Clearly, treatment results of studies encompassing small sample sizes (*n* < 100) must be considered with particular caution since chance results are likely. Therefore, to allow for a balanced and meaningful discussion of results, we excluded studies with ≤ 50 patients. Additionally, pertinent large-scale studies of surveillance in GCT CSI patients were additionally identified from electronic databases. These data were listed in separate tables [19–22] and then employed in the discussion along with the data of the primary search. Although in the primary literature search several duplicate studies were excluded (Fig. 1), there is still a chance that a number of cases might have been included in more than one study. However, we are not aware of unequivocal evidence for duplicate publication among the studies included in the analysis.

### Non-seminoma

The crucial result regarding non-seminoma patients is that the overall survival is close to 100% for all post-orchiectomy treatment modalities. Surveillance in NS patients without considering risk factors resulted in relapse rates of 27.2–37% reported in large-scale studies. The amazingly low rate of 19% reported from the Canadian study does probably relate to the very low proportion of high-risk patients (16%) [20].

Histologic evidence of lymphovascular invasion (LVI) is the most widely adopted tool for identifying patients at high risk of progression [23]. Patients with this histologic feature develop relapses upon surveillance in 42–78% of cases [22, 24], while patients without LVI will relapse in 13–25% [16, 24]. These data provide evidence for the principal usefulness of LVI to indicate a high risk of progression, which is in accordance with previously published data [25]. But clearly, recurrence rates of up to 25% despite the absence of LVI indicate that this factor represents a diagnostic tool with only little sensitivity and specificity. Accordingly, more sensitive factors indicating the risk of recurrence are urgently needed.

Adjuvant chemotherapy with one or two cycles of cisplatin, etoposide and bleomycin (BEP) significantly reduces the risk of relapse to less than 5% as found in the present analysis (Table 10). A recent meta-analysis of adjuvant chemotherapy in NS CSI [26] reported a relapse of 1.8% following the two cycle BEP regimen and a rate of 2,3% with respect to 1 course BEP [26]. A sub-standard chemotherapy regimen employing vinblastine instead of etoposide (PVB) yielded inferior results [16]. RPLND is currently only recommended in selected cases of NS CSI. Therefore, only two small studies were identified during the search period of this review. The reported relapse rates of 0–11.8% are in line with results reported from traditional large-scale series [4, 25, 27]. However, comparison is hampered by selection bias among studies and by lacking information about additional chemotherapy in pathological stage II cases.

**Table 10:** Relapse rates (%) in non-seminoma CSI patients managed by adjuvant chemotherapy

First author	Year	Design	Chemotherapy protocol	Study Period	*n*	Relapse rate
Dieckmann [13]	2018	Retrospective cohort study	2 × BEP	2008–2017	54	0%
Ondrusova [14]	2017	Retrospective cohort study	2 × BEP	1992–2017	184	1.1%
Ondrus [15]	2015	Prospective cohort study	2 × BEP	1992–2014	167	1.2%
Kobayashi [12]	2013	Retrospective cohort study		1980–2008	4	0%
Lv [16]	2013	Retrospective cohort study	2 × BEP	1997–2011	18	5.6%
Tandstad [17]	2010	RCT	2 × PBV* 1 × PBV	1995−1998	103	High risk: 1.6% Low risk: 10% unclear: 0%

*Vinblastine; this regimen is no longer standard treatment

Salvage therapy of relapses involving cisplatin-based chemotherapy and additional residual mass resections is efficacious in the vast majority of cases. Thus, the high relapse rates of up to 60% documented in some studies [16] do not translate into inferior overall survival of patients undergoing surveillance. Accordingly, OS rates of 94.2 to 100% were reported for patients on surveillance from comparative studies [13, 28], which are confirmed with the rates of 96.2 and 99.4% observed in two large multicentric single-arm studies from Denmark and Canada (19), (21). Noteworthy, a retrospective analysis of the National Cancer Database of the US revealed slightly higher 5-year OS rates compared to 10-year survival rates which probably reflects the occurrence of competing reasons for death over time. Moreover, that analysis revealed slightly higher OS rates in CSIa than in CSIb of 97.3 versus 96.5%. Although this difference is very small, it might reflect the experience that isolated cases with relapses might fail salvage treatment which is the experience in all major series [19, 20, 22].

The OS rates achieved with adjuvant treatment modalities are not truly different from those of surveillance (Tables 3, 4), ranging from 91.7 to 100% in adjuvant chemotherapy [13, 28] and 97.0–99.1% in RPLND [28].

Overall, these results indicate the non-inferiority of surveillance to adjuvant management modalities and clearly provide again justification for the surveillance strategies. However, as salvage therapies required in recurrences may involve considerable treatment-related toxicity, patients with high risk of progression (LVI) may benefit from adjuvant treatment that is usually well-tolerated and associated with only little long-term sequelae [29]. Finally, a shared-decision-making process is recommended to reach an appropriate treatment decision [7].

### Seminoma

#### Surveillance

The currently most widely adopted management modality in SE CSI patients is surveillance [17]. The present analysis revealed relapse rates in SE patients of 7%–22.3% when no risk factors are considered [30–32] which is consistent with relapse rates of 13–18.8%. reported from large-scale single-arm studies [33, 34]. Primary tumour size > 4 cm is the only recognized factor that may identify patients at higher risk of recurrence [10]. In fact, several studies showed significantly higher recurrence rates in patients with tumours sized > 4 cm [22, 31, 35, 36], usually in the range of 15–18%. However, other studies did not reveal differences [17], and even in low-risk cases recurrences were observed in 4–14% [31, 35]. Methodologically, the discrimination between high risk and low risk of recurrence of seminomas indicated by tumour size is much less feasible than separating high risk of recurrence from low risk in non-seminomas with the factor LVI. Thus, tumour size though easy to apply practically, is apparently of limited utility.

### Carboplatin chemotherapy versus surveillance

There are no prospective randomized controlled trials comparing carboplatin monotherapy with surveillance in CSI SE patients. Survival data and recurrence rates after adjuvant carboplatin therapy are only available from multi-arm prospective cohort studies and from a large number of retrospective single-arm studies.

Concerning disease free survival, the 3 years’ disease free rate after two courses of carboplatin monotherapy is 98.0% (95% CI 94.0–100%) [30]. These results were widely confirmed in an extended study of the same authors 3 years later [31]. The reported studies uniformly show lower recurrence rates of 0.0–5.2% subsequent to carboplatin compared to 8.2–22.3% in patients undergoing surveillance [32, 35, 37–39].

The question whether two cycles of carboplatin result in lower relapse rates compared to the one course regimen has not been systematically evaluated. However, three studies indicated relapse rates of > 10% ensuing from the one course regimen [37, 40, 41], while all single-arm studies employing the two-course regimen reported relapse rates < 5%. On the other hand, a worse outcome was observed in cases relapsing after two cycles of carboplatin, although some selection bias must be considered in that retrospective study [42]. In conclusion, the superiority of two courses carboplatin over one remains unclear.

Importantly, the lower recurrence rates after adjuvant carboplatin therapy did not translate into superior survival rates (overall and cancer-specific) compared to surveillance. The 5-year overall survival rates range from 98.9–99.2% and 98.4–99.2% for carboplatin and surveillance, respectively [35, 40], while the 10-year OS rates are 98.5 and 96.8% for patients treated with carboplatin and those under surveillance, respectively [40].

Survival rates could be confounded by a selection bias in the two SWENOTECA studies [35, 40], because patients were able to choose their own treatment modality, even against the recommendation of the treating physicians. For example, in the Tandstad 2016 study, only 11% of the included patients had indeed two risk factors qualifying for carboplatin therapy according to the protocol, and 53% of the patients opting for carboplatin therapy were without risk factors. Thus, carboplatin therapy might have yielded inappropriately favorable results because of a confounded patient sample.

### Adjuvant radiotherapy versus surveillance

There are no prospective RCTs directly comparing the efficacy of surveillance and adjuvant retroperitoneal irradiation for CSI seminoma patients.

A retrospective, two-armed cohort study compared the efficacy of surveillance to adjuvant radiotherapy in 473 seminoma patients with a primary tumour size of ≥ 6 cm [34]. Radiotherapy reduced the incidence of relapse from 32 to 2.8% after 10 years, but there were no statistically significant differences in 10-year OS (92.4% surveillance versus 94.2% irradiation). Importantly, also the incidence of subsequent malignant neoplasms was not significantly different between the two strategies with a 15-year incidence of 6.5% in surveillance patients and 9.9% in those with irradiation.

Several retrospective studies confirmed the significantly lower relapse rates in irradiated patients than in those on surveillance [32, 43, 44], and also the non-divergent rates of subsequent malignant neoplasms [45].

In a Cox regression analysis, radiotherapy was found to result in the lowest relapse rates among all adjuvant management strategies [35].

The American SEER database, including 6764 patients treated between 1973 and 2003, showed a better OS for radiotherapy than for surveillance after an average of 7.6 years, but a worse after 20 years. Cancer-specific survival was higher in the radiotherapy group after 5, 10 and 20 years than in the surveillance group. While these results appear remarkable at first glance, there are three major concerns relating to this work. 1) There was a large difference in subgroup sizes with over 5265 irradiated patients compared to 1499 patients under surveillance. 2) There is no information regarding the radiation dose and field size. 3) The median follow-up period was only 7.6 years, which is clearly too short to reveal the rate of secondary malignancies, as the incidence is increasing after more than 10 years [18].

### Adjuvant radiotherapy versus carboplatin versus surveillance

Studies comparing all three treatment options, showed equivalent efficacy of adjuvant radiotherapy and one cycle of carboplatin administration with respect to 5-year recurrence rates (2–2.4% in radiotherapy versus 2–5% after carboplatin) [37, 44] but other studies documented superiority of radiotherapy over carboplatin with regard to 5-year recurrence rate (0.8 versus 3.9%; *p* = 0.03) [35]. The 5-year recurrence rates after surveillance were 8.2, 19.7, and 14.3% in three studies directly comparing all three management modalities [35, 37, 44] thus being significantly higher than after any adjuvant therapy. However, overall and disease-specific 5 years’ survival was not different between the treatment strategies. [35].

Some distinct advantages may result from active surveillance. Terbuch et al. [46] evaluated the long-term consequences of 406 seminoma patients managed between 1994 and 2013. Noteworthy, the risk of cardiovascular diseases was significantly increased after radiotherapy. Similarly, active surveillance was found to be significantly better tolerated than carboplatin therapy as reported from a retrospective study on 451 seminoma patients treated in Germany from 1994 to 2014 [47].

A large Danish retrospective cohort study including 5,190 seminoma and non-seminoma patients treated between 1984 and 2007 investigated the therapy-related risk of second malignant neoplasms after active surveillance, chemotherapy (with bleomycin, etoposide and cisplatin) or radiotherapy. Importantly, the authors found that, except for active surveillance, all other treatment modalities involved a dose-dependent increased risk of a second malignancy [48].

## Conclusion

Active surveillance is a safe treatment option in non-metastasized GCTs that is usually well tolerated by the patients. Although it initially results in higher rates of recurrence compared to interventional adjuvant strategies, overall survival rates are not different among the various treatment modalities. Almost all of the relapsing patients can be successfully salvaged with stage adjusted treatment.

In high-risk non-seminoma, the rate of relapses is about 50% in patients on surveillance. Applying one cycle of adjuvant chemotherapy in all high-risk non-seminoma CSI may reduce the total number of chemotherapy cycles needed in the entire population of these patients if balanced against the total number of cycles required for those on upon salvage therapy. Although the evidence is still limited to date [29], the overall burden of long-term toxicity may be lower in high-risk CSI non-seminoma patients receiving adjuvant therapy than in those on surveillance. In conclusion, while there are several treatment modalities of equivalent efficacy available in GCT CSI, the optimized management needs to be tailored to the individual needs of each patient.

## Supplementary Information

Below is the link to the electronic supplementary material.

Supplementary file1 (DOCX 91 KB)

Supplementary file2 (DOCX 28 KB)
