# Supplementary material for: Testicular germ cell tumours’ clinical stage I: comparison of surveillance with adjuvant treatment strategies regarding recurrence rates and overall survival—a systematic review
Source: World J Urol. 2022 Sep 15;40(12):2889–900. doi: 10.1007/s00345-022-04145-6 (PMC9712330; doi:10.1007/s00345-022-04145-6)
Supplement: Supplementary file 1 — Supplementary file1 (DOCX 91 KB) [file 345_2022_4145_MOESM1_ESM.docx]

**Supplementary table 1**

| Reference | **Design**  **Number of patients**  **Country**  Follow-up | Objective | Patients | Intervention | Control | Results | **Funding and conflict of interests** | **Level of evidence**  **Risk of bias**  *additional comments* |
| --- | --- | --- | --- | --- | --- | --- | --- | --- |
| Aparicio  2011 | prospective cohort study  n=227  2004-2008  Spain  Median follow-up:  34 mo | To reduce both the risk of relapse and the proportion of patients receiving  adjuvant chemotherapy while maintaining a high cure rate. | clinical stage I seminoma  Mean age: 33 y (21-59 y) | Surveillance  n=153 | Carboplatin  n=74 | **Surveillance**  3-y DFS   - total: 88.1% (95% CI 82.3%-93.9%) - no risk factors: 93.5% - with tumour size > 4 cm: 83.7% - with rete testis involvement: 78.3%   3-y OS  100%  **Adjuvant chemotherapy**  3-y DFS  total: 98.0% (95% CI 94.0%-100%)  3-y OS  100%  Adverse events  - uncomplicated thrombocytopenia (8%)  - afebrile neutropenia (4%),  - anemia (2%),  - emesis (2%) | No information on sources of funding. | LoE 2b  RoB:  acceptable  No indication for comparison between full participants and those lost to follow-up;  No mention of confounding, descriptive comparison |
| Aparicio  2014 | retrospective cohort study  n=744  1994-2008  Spain  Median follow-up time from orchiectomy:  80 mo (range, 24–204 mo) | We aimed to analyze prognostic factors for relapse in stage I seminoma managed by either active surveillance  or adjuvant chemotherapy, and to describe the long-term patterns of recurrence in both groups. | clinical stage I seminoma | Low-risk:  Surveillance | High-risk:  two courses of adjuvant carboplatin | **Relapse**  Surveillance: 51/396 (14.8%)  Carboplatin: 12/348 (3.2%)  **5-y DFS**  Surveillance: 88.3%  Carboplatin: 96.8%  p=0.0001  **10-y DFS**  Surveillance: 85.2%  Carboplatin: 96.8%  p=0.0001 | The authors have declared no conflict of interest. | LoE 4  RoB: not acceptable  Confounding is mentioned, but no comment or analysis of potential impact on results.  No confidence intervals given;  Groups have different risk profiles. |
| Aparicio  2018 | prospective cohort study  n=135  2013-2015  Spain  Median follow-up:  33 mo (12-57 mo)  78% had a follow-up over 24 mo and 43% over 36 mo | The aim of this study was to assess a risk-adapted strategy for stage I seminoma guided by the presence of rete testis invasion. | clinical stage I seminoma  Age: 38 y (22-60 y)  Median tumor size: 4 cm (0.4-14 cm) | Surveillance  n=71 | Carboplatin  n=64 | **Number of relapses**  Surveillance: 5/71  Carboplatin: 1/64  **3-y DFS**  Overall: 94.9% (95% CI 90.7-99.1%)  Surveillance: 92% (95% CI 85-99%)  Carboplatin: 98.2% (95% CI 94.6-100%)  p=0.139  **3-y OS**  100% | The authors have no conflicts of interest to disclose. | LoE 4  RoB: not acceptable  No information on age; No indication of the actual participation rate; There is no comparison between full participants and those lost to follow up; No mention of confounding. |
| Bilici  2015 | retrospective cohort study  n=282  1997-2013  Turkey  Median follow up:  38.5 mo  (6.5-192 mo) | To evaluate  treatment preference and outcomes for men with stage I testicular seminoma. | clinical stage I seminoma  Median age total group: 35 y  Surveillance: 33.5 y (19-85 y)  Carboplatin: 33.5 y (18-64 y)  Radiotherapy:  36 y (17-74 y) | Surveillance  n=72 | Radiotherapy  n=130  Carboplatin  n=80 | **Relapse Rate**  Surveillance: 22.3%  Carboplatin: 1.2%  Radiotherapy: 7.7%  p<0.001  **5-y DFS**  Surveillance: 64.2%  Carboplatin: 97.7%  Radiotherapy: 91.9%  p<0.001  **5-y OS**  Surveillance: 100%  Carboplatin: 92.3%  Radiotherapy: 97.4%  p=0.44 | no financial support  The authors declare that they have no  conflict of interest. | LoE 2b  RoB:  acceptable  No indication if or how measures were managed (regarding detection bias in outcome assessment);  No mention of confounding |
| Dieckmann 2013 | retrospective cohort study  n=228  Germany,  Austria  2006-2010  Median follow-up of all patients:  4 y (range 0.1–13.1 y) | The aim of the present study is to evaluate the effects of  radiotherapy and chemotherapy on biopsy-proven testicular intraepithelial neoplasia in a  large cohort of patients. | patients with unilateral germ cell tumors and biopsy-proven contralateral testicular intraepithelial neoplasia  n=116 non-seminoma  n=112 seminoma  Mean age:  30.5 y (SD 6.9 y) | Surveillance  n=10 | Local radiotherapy  n=122  Cisplatin-based chemotherapy (two cycles)  n=30  Chemotherapy (three cycles)  n=51  Carboplatin  n=15 | **Malignant events**  Surveillance: 5 (50%)  Radiotherapy: 3 (2.5%)  Chemotherapy (2 cycles): 15 (50%)  Chemotherapy (3 cycles): 12 (23.5%)  Carboplatin: 10 (66%)  **Event-free survival**  Surveillance: 7.08 y  Radiotherapy: 11.8 y  Chemotherapy (2 cycles): 3 y  Chemotherapy (3 cycles): 9.83 y  Carboplatin: 0.9 y  **HR of developing malignant event**  Surveillance: 12.3 (95% CI 2.88-52.35)  Radiotherapy: 1  Chemotherapy (2 cycles): 29.1 (95% CI 8.41–100.85)  Chemotherapy (3 cycles): 11.5 (95% CI 3.15–42.30)  Carboplatin: 63.6 (95% CI 17.21–235.11)  **Hypogonadism rate**  Surveillance: 40% (95% CI 12.16–73.76)  Radiotherapy: 30.8% (95% CI 22.41–39.13)  Chemotherapy (2 cycles): 13% (95% CI 2.78–33.59)  Chemotherapy (3 cycles): 17.8% (95% CI 8.0–32.05)  Carboplatin: 40% (95% CI 12.16–73.76) | The authors have declared no conflicts of interest.  no information about funding | LoE 2b  RoB:  acceptable |
| Dieckmann 2016 | prospective cohort study  n=725  2008-2013  Germany  Median follow up:  30 mo (0-60 mo) | We evaluated  the relapse rates in the four management modalities,  and we also looked at the significance of risk factors with  respect to disease progression. | clinical stage I seminoma  Mean age  Surveillance: 40 y (20-75 y)  Radiotherapy: 39 y (25-65 y)  1x Carboplatin: 42 y (19-82 y)  2x Carboplatin: 43.5y (21-81 y) | Surveillance  n=256 | Radiotherapy  n=41  Carboplatin  (1 course)  n=362  Carboplatin  (2 course)  n=66 | **Relapse rate**  Surveillance: n=21 (8.2%)  Radiotherapy: n=1 (2.4%)  1x Carboplatin: n=18 (5%)  2x Carboplatin: n=1 (1.5%)  **Dead without disease**  Surveillance: 0  Radiotherapy: 0  1x Carboplatin: n=2 (0.6%)  2x Carboplatin: n=0  **Association with relapse**  Rete testis invasion and tumor size were not associated with progression in surveillance patients.  1x Carboplatin  tumor size <4 cm: 2.3%  tumor size: >4 cm: 6.8%  p=0.04  tumor size >4 cm vs tumor size <4 cm:  HR 3.03 (95% CI 0.97-9.44) | Funding source: Hamburger Stiftung zur Förderung der Krebsbekämpfung  The authors declare no conflicts of interests with the article. | LoE 2b  RoB:  acceptable  No indication of dropout rates;  No mention of confounding |
| Dieckmann  2018 | retrospective cohort study  n=75 consecutive patients  2008-2017 | We evaluated the practice patterns of the management of clinical stage 1 patients and looked specifically to the role of retroperitoneal lymph node dissection among other  standard treatment options. | clinical stage I  non-seminomatous testicular germ-cell tumor | Surveillance  n=9 | nerve-sparing RPLND  n=12  adjuvant chemotherapy  n=54 | Relapse  Surveillance: 2/9  RPLND: 0/12  Adjuvant chemotherapy: 0/54 | This study did not receive any funding.  The authors declare that they have no competing interests. | LoE 4  RoB:  not acceptable  Source populations are identified but no specific characteristics are tabulated; Outcomes and measurement methods are not discussed; No mention of confounding; No confidence intervals provided. Descriptive statistics only. Mainly describing patients undergoing primary RPLND.  *Median follow-up not reported* |
| Dong 2013 | Retrospective cohort study  n=195  China  1997-2009  Median follow-up: 82 mo (18-195 mo) | We aimed to investigate predictors for longterm  outcomes of surveillance, RPLND, and primary chemotherapy  following orchiectomy in patients with clinical stage I NSGCT and to explore the high-risk factors for relapse in  surveillance group. | patients with clinical stage I  NSGCT  n=89 clinical stage 1 tumors  Mean age  Surveillance: 18.4 y (3–46 y)  RPLND: 29.7 y (13–47 y)  Chemotherapy: 33.0 y (8–66 y) | Surveillance  n=38 | Chemotherapy  n=21  RPLND  n=30 | **Tumor relapse**  Surveillance: 8/38  Chemotherapy: 1/21  RPLND: 0/30  **Cumulative 5-y PFS rates**  Surveillance: 74.1%  Chemotherapy: 92.3%  RPLND: 100%  p=0.013  **5-yDSS**  All groups: 100%  **5-y OS**  All groups: 100% | No information about funding  All authors declare that there is no conflict of interest. | LoE 2b  RoB: acceptable  The mean age of the groups and the histology of primary lesion differs significantly. |
| Gumus 2017 | Retrospective cohort study  n=201  Turkey  1999-2013  Median follow-up: 34 mo (8-162 mo) | The purpose of this study was to compare outcomes of active surveillance with adjuvant chemotherapy. | patients with clinical stage I NSGCT  Median age: 28 y (16-59 y) | Surveillance  n=91 | Adjuvant chemotherapy  n=110 | **Relapses**  Surveillance: 17/91 (18.3%)  Chemotherapy: 1/110 (1.2%)  p<0.001  **RFS rate**  Surveillance: 80.8%  Chemotherapy: 97.6%  p<0.001  Treatment strategy was an independent prognostic factor for RFS (p<0.001, HR 0.54).  **5-y OS**  Surveillance: 96.1%  Chemotherapy: 100%  p=0.12 | Not supported by any financial  or other relationships.  The authors declare that they have no conflict  of interest. | LoE 2b  RoB: acceptable  Endpoints were not pre-defined. |
| Haugnes 2014 | retrospective cohort study  n=232  1986-2010  Norway | The aim of the present study was to present the results of  seminoma treatment with respect to stage, i.e clinical stage I vs.  metastatic disease, and treatment period, before vs. after the  SWENOTECA V guidelines. | n=198 clinical stage I seminoma  Median age:  38 y (24-77 y) | Surveillance  n=73 (66%) | Radiation  n=23 (21%)  Carboplatin:  n=15 (13%) | **Time to relapse**  all: 15 mo (4-93 mo)  **Relapse-free survival**  all: 93%  **Relapse rate:**  radiation: 1.9%  carboplatin: 0%  surveillance: 11%  **Cancer-specific survival** all: 100% | no information about conflict of interest  Norwegian Cancer Society supported this project | LoE 2b  RoB: acceptable  No indication if or how measures were managed (regarding detection bias in outcome assessment);  No mention of confounding;  No confidence intervals given (descriptive comparison)  *Median follow-up not reported* |
| Jones  2013 | retrospective cohort study  n=6764  1973-2003  USA  Median follow-up  Radiation: 96 mo (0-354 mo)  Observation: 78 mo (0-340 mo) | We analyzed  survival rates in men with stage I seminoma who underwent  adjuvant radiation therapy or observation after orchiectomy. | clinical stage I seminoma  n=6764  Mean age: 36.6 (9.58 y) | Surveillance  n=1499 | Radiation  n=5265 | **Overall survival**  Surveillance  5-y OS: 95.0%  10-y OS: 92.2%  20-y OS: 84.1%  Radiation  5-y OS: 97.7%  10-y OS: 94.8%  20-y OS: 83.5%  p=0.0047  **Cause specific survival**  Surveillance  5-y CSS: 98.7% (98.1-99.4)  10-y CSS: 98.7% (98.1-99.4)  20-y CSS: 98.7% (98.1-99.4)  Radiation  5-y CSS: 99.6 (95% CI 99.4-99.8)  10-y CSS: 99.4 (95% CI 99.2-99.7)  20-y CSS: 99.2 (95% CI 98.8-99.6)  Surveillance vs. Radiation: p=0.0015  **Freedom from second malignancy diagnosis**  Surveillance  5-y: 98.5 (95% CI 97.8-99.2)  10-y: 97.3 (95% CI 96.2-98.4)  20-y: 95.0 (95% CI 92.9-97.1)  Radiation  5-y: 97.7 (95% CI 97.3-98.2)  10-y: 95.8 (95% CI 95.1-96.5)  20-y: 87.9 (95% CI 86.0-89.8)  p=0.0029  **Freedom from third malignancy diagnosis**  Surveillance  5-y: 95.5 (95% CI 87.1-100)  10-y: 95.5 (95% CI 87.1-100)  20-y: 70.7 (95% CI 45.7-100)  Radiation  5-y: 99.2 (95% CI 98.1-100)  10-y: 97.8 (95% CI 95.6-100)  20-y: 76.1 (95% CI 65.0-89.0)  p=0.2669 | The authors declare no conflicts of interest  no information about Funding source | LoE 2b  RoB:  acceptable  No indication if or how measures were managed (regarding detection bias in outcome assessment);  No mention of confounding |
| Kamba  2010 | retrospective cohort study  n=425  1985-2006  Japan  Median follow up:  Surveillance:  44.9 mo (0.1–218.7 mo)  Chemotherapy:  58.4 mo (2.5–205.6 mo)  Radiation  60.8 mo (0.9–248.5 mo) | To clarify the contemporary clinical outcome of stage I seminoma and to provide information on treatment  options to patients. | clinical stage I seminoma  Median age  36 y (19-84 y) | Surveillance  n=186 | Radiation  n=182  Chemotherapy  n=57 | **Median time for relapse**  Surveillance: 21.0 mo Radiotherapy 37.9 mo  Chemotherapy 42.8 mo  **10-y OS**  Surveillance: 100%  Radiotherapy: 99.4%%  Chemotherapy: 100%  **5-y RFS**  Surveillance: 90%  Radiotherapy: 95%  Chemotherapy: 94%  **10-y RFS**  Surveillance: 79%  Radiotherapy: 94%  Chemotherapy: 94%  RFS was significantly better in the chemotherapy and radiotherapy groups than in the surveillance  group (p=0.0201) | There are no conflicts of interest.  no information about funding source | LoE 2b  RoB:  acceptable  No indication if or how measures were managed (regarding detection bias in outcome assessment);  No mention of confounding;  No confidence intervals given |
| Kamran  2018 | retrospective cohort study  n=1362  2004-2012  USA  Follow-up:  Seminoma  51 mo (31-73 mo)  Non-seminoma  49 mo (27-72 mo) | To assess contemporary treatment patterns and outcomes for clinical stage IS  testicular cancer. | clinical stage IS  testicular cancer  n=581 seminoma  n=781 non-seminoma  Mean age  Seminoma: 73.3 y (SD 9.9 y)  Non-seminoma: 30.5 y (SD 9.4 y) | Initial surveillance  n=638 | Adjuvant treatment  n=724 | Seminoma  Deaths  Surveillance: 8/227  Adjuvant treatment: 5/354  5-y OS  Surveillance: 99%  Adjuvant treatment: 99%  p=0.08  Mortality  HR 0.36 (95% CI 0.12-1.14) p=0.08  Non-seminoma  Deaths  Surveillance: 14/411  Adjuvant treatment: 9/370  5-y OS  Surveillance: 95%  Adjuvant treatment: 97%  p=0.36  Mortality  HR 0.66 (95% CI 0.27-1.61) p=0.36 | Honoraria: National  Comprehensive Cancer Network, UpToDate Consulting, or Advisory Role; Pfizer, Bayer AG, Novartis, GlaxoSmithKline, Merck, Bristol-Myers Squibb, Genentech, Eisai, Prometheus Labs, Foundation Medicine  Research, Cerulean Pharma, AstraZeneca, Peloton  Funding: Pfizer (Inst), Novartis  (Inst), Merck (Inst), Exelixis (Inst), TRACON Pharmaceuticals (Inst),  GlaxoSmithKline (Inst), Bristol-Myers Squibb (Inst), AstraZeneca (Inst),  Peloton Therapeutics (Inst), Genentech (Inst). | LoE 2b  RoB:  acceptable  “[…] complete information on serum tumor marker absolute values before and after radical inguinal orchiectomy was unavailable”  “[…] the National Cancer Database does not provide detailed information on chemotherapy regimens, cycles, nor radiation therapy techniques.” |
| Khader  2012 | retrospective cohort study  n=74  2003-2010  Jordan  Mean follow-up: 33 mo | To address treatment  outcomes in patients with early-stage seminoma in a  developing country with special reference to patients who  underwent surgical violation of the scrotum. | clinical stage I seminoma  Mean age:  34 y (17–51 y) | Surveillance  n=3 | Radiation n=71 | **Relapses**  Surveillance: 0/3  Radiation: 3/71  3-y relapse-free survival (entire cohort): 95.9% | The authors declare that they have no competing interests.  no information about funding source | LoE 4  RoB:  not acceptable  Outcomes aren’t defined  No indication if or how measures were managed (regarding detection bias in outcome assessment);  No mention of confounding;  No confidence intervals given, descriptive comparison |
| Kier  2016 | retrospective cohort study  n=5190  1984-2007  Denmark  Mean follow-up: 14.4y  (8.6y – 20.5y) | To evaluate the treatment-specific risks for second malignant neoplasm and death in a nationwide population-based cohort of patient with germ cell cancer treated with current standard regimes. | stage I germ cell tumor  n=2804 patients with seminoma  n=2386 patients with non-seminoma | Surveillance  n=335 | Chemotherapy (1x BEP)  n=1862  retroperitoneal Radiation  n=787  more than one line of treatment  n=304 | **Second malignancies at 20 y**  Surveillance: 7.8%  BEP: 7.6%  Radiotherapy: 13.5%  more than one line of treatment: 9.2%  **Risk for second malignancies**  Surveillance: HR 1  BEP: HR 1.7 (95% CI 1.4-2)  Radiotherapy: HR 1.8 (95% CI 1.5-2.3)  more than 1 line of treatment: HR 3.7 (95% CI 2.5-5.5)  **20-y probability of death**  Surveillance: 9.3%  BEP: 13.6%  Radiotherapy: 14.7%  more than 1 line of treatment: 74.5% | Danish Cancer Society  and several more foundations  no information about conflict of interests | LoE 2b  RoB:  acceptable  No indication if or how measures were managed (regarding detection bias in outcome assessment);  No mention of confounding  *no information about age* |
| Kobayashi 2013 | retrospective cohort study  n=158  1980-2008  Japan  Mean follow-up:  Surveillance: 67 ± 50  Radiation: 174 ± 54 | This study was performed to explore pathological risk factors for  post-orchiectomy relapse in patients with stage I seminoma and non-seminoma, and to assess oncological  outcomes in those managed with surveillance. | n=118 stage I seminoma  n=40 stage I non-seminoma  Mean age:  37.0 ± 10.6 | **Surveillance**  n=36 patients with non-seminoma  n=61 patients with seminoma | **Chemotherapy**  n=4 patients with non-seminoma  **Radiation**  n=56 patients with seminoma | **Relapse rate**  Seminoma patients  Surveillance: 4 (6.6%)  Radiation: 1 (1.7%)  p=0.19  **10-y relapse-free survival**  Surveillance: 93.4%  Radiation: 98.2%  p=0.15  Non-seminoma patients  Surveillance: 9 (25%)  Chemotherapy: 0  **5-y/10-y relapse-free survival:**  surveillance: both 75% | not been funded by any commercial company or grant  The authors declare that they have no competing interests. | LoE 4  RoB:  not acceptable  No definition of outcomes.  No indication if or how measures were managed (regarding detection bias in outcome assessment);  No mention of confounding  *no information about relapse-free survival in the chemotherapy group* |
| Kollmanns-berger  2011 | retrospective cohort study  n=649  1999-2008  Canada  Median  follow-up  Surveillance: 34 mo (2–136 mo)  Radiation:  65 mo (3–120 mo)  Carboplatin  33 mo (4–106 mo) | We report the evolution of treatment with increased use of active surveillance for stage I  disease as well as risk-adapted chemotherapy for disseminated disease and associated outcomes of testicular  seminoma in a contemporary population-based cohort. | clinical stage I seminoma  n=545  Mean age:  Surveillance: 37 y (18–83 y)  Radiation  39 y (19–76 y)  Carboplatin  36y (19–62y) | Active surveillance  n=313 | Adjuvant radiotherapy  n=159  Adjuvant carboplatin  n=73 | **5y relapse-free survival**  Surveillance: 80.7%  Radiotherapy: 98%  Carboplatin: 98%  Log rank p<0.001  **Dead of disease**  Surveillance: 0  Radiotherapy: 0  Carboplatin: 0 | The authors declare no conflict of interest.  no information about funding source | LoE 2b  RoB: acceptable  No indication if or how measures were managed (regarding detection bias in outcome assessment);  No mention of confounding; No confidence intervals given |
| Leung  2013 | retrospective cohort study  n=764  1981-2004  Canada  Median follow-up  Surveillance: 6.6 y  Radiotherapy: 8.5 y | To examine the management and outcomes of patients with  stage I seminoma and to relate these to overall treatment  burden. | clinical stage I seminoma | Surveillance  n=484 | Radiation  n=280 | **Surveillance:**  5-y OS: 98.6%  10-y OS: 97.7%  n=72 (15%) relapsed  median time to relapse: 14 mo  **Adjuvant Radiotherapy:**  5-y OS: 97.2%  10-y OS: 91.4%  n=14 (5%) relapsed  median time to relapse: 15 mo | The authors declare no conflict of interest.  no information about funding source | LoE 2b  RoB: acceptable  No indication if or how measures were managed (regarding detection bias in outcome assessment);  No mention of confounding;  No confidence intervals given (descriptive comparison)  *no information about age* |
| Lv  2013 | retrospective cohort study  n=89  1997-2011  China  Median follow-up:  92 mo  (6-149 mo) | This study evaluates the long-term outcomes of the applied treatment  strategies of surveillance, RPLND and adjuvant chemotherapy in Han Chinese patients. | clinical stage I non-seminomatous  germ cell tumour  Mean age: 26.26±13.4y | Active surveillance:  n=37 | RPLND:  n=34  Adjuvant chemotherapy  n=18 | **4-y recurrence-free rate**  Surveillance (low-risk-patients): 80.2%  RPLND (low and high risk patients): 92.0%  Adjuvant chemotherapy (low and high risk patients): 100%  p=0.441  **Relapses**  Surveillance: 8/37 (21.6%)  RPLND: 4/34 (11.8%)  Adjuvant Chemotherapy: 1/18 (5.6%) | four public funding sources  All authors declare that there are no competing financial interests. | LoE 2b  RoB: acceptable  Confounding is mentioned, but no comment or analysis of potential impact on results. |
| Mahantshetty 2012 | retrospective cohort study  n=137  1990-1998  India  Median follow up  Radiation: 34 mo  Surveillance: 29 mo | With an aim to introspect the outcome of patients with stage I seminoma, we undertook a retrospective analysis and form the basis of the report. | clinical stage I seminoma  mean age: 37 y (20-68 y) | Surveillance  n=41 | Radiation  n=96 | **5-y DFS**  Surveillance: 73.5%  Radiation: 91%  p=0.004  **5-y DSS**  Surveillance: 89%  Radiation: 93%  p=0.18  **Late Grade III toxicity**  n=0 in both groups  **Second cancer**  n=0 in both groups | no information about conflict of interests and about funding source | LoE 4  RoB: not acceptable  Follow-ups in abstract are different from follow-up in full text;  Outcomes aren’t defined;  No indication if or how measures were managed (regarding detection bias in outcome assessment);  No mention of confounding;  No confidence intervals given |
| Mistretta  2019 | retrospective cohort study  n=11206 patients  1988-2015  USA | We postulated that surveillance does not result in inferior CSM or OCM, relative to alternative management strategies.  To validate this hypothesis, we tested for differences in CSM  according to management strategy type with adjustment for OCM,  using multivariable propensity score-adjusted competing risks  regression models. | stage I testicular seminoma patients  Median age  Surveillance: 36 y  Active treatment: 37 y | Surveillance | Active treatment (chemotherapy/radiotherapy) | **Surveillance vs. Active treatment**  surveillance was an independent predictor of CSM (HR 2.59 (1.06-6.32) p=0.04)  did not affect OCM (HR 1.52 (1-2.32), p=0.051)  **Surveillance vs. radiotherapy**  no significant differences  **Surveillance vs. chemotherapy**  no significant differences  **Radiotherapy vs. chemotherapy**  no significant differences | The authors have stated that they have no conflicts of interest. | LoE 2b  RoB: acceptable  The performance of interventions are not described.  *Median follow-up not reported* |
| Ondrus  2015 | prospective cohort study  n=454  January 1992 – August 2014  Slovakia | The aim of contemporary study was to  correlate own long-term experiences with  active surveillance and adjuvant chemotherapy,  resp. in clinical stage I non-seminomatous germ cell testicular tumors patients. | Non-seminomatous germ cell testicular tumors I  low risk n=287  (negative LVI)  high risk n=167  (with LVI and/ or > 50% embryonal cell carcinoma) | Active surveillance  n=287 low risk patients | adjuvant Chemotherapy (two cycles BEP)  n=167 high risk patients | **Relapse**  Surveillance  48/287 (16.7%)  median follow-up of 7.0 mon  Chemotherapy  2/167 (1.2%)  median follow-up of 56.2 mo  **PFS**  Surveillance  83.3%  median follow-up 113.9 mo  Chemotherapy  98.8%  median follow-up of 134 mo  p<0.001  **OS**  Surveillance  281/ 287 (97.9%)  with median  follow-up of 142 mo  Chemotherapy  166/ 167 (99.4%)  median follow-up of 135.7 mo  No significant difference  in OS was recorded. | supported  by the Slovak Research and Development  Agency.  The authors declare they have no potential  conflicts of interest concerning drugs,  products, or services used in the study. | LoE 4  RoB: not acceptable  Authors do not indicate the actual participation rate.  Outcome Survival is not defined.  No mention of confounding;  No confidence intervals given  *different follow-up periods for treatment groups and PFS, OS* |
| Ondrusova  2015 | retrospective cohort study  n=90  2008-2015  Slovakia  Mean follow-up for OS:  27 mo (6.5–84 mo) | We analyzed single center  experience with risk-adapted therapeutic approaches  (active surveillance and adjuvant chemotherapy) in patients  with clinical stage I seminomatous  germ cell testicular tumors. | clinical stage I seminoma  Mean age:  36.6 y | Surveillance  (low risk group)  n=74 | Carboplatin  (high risk group)  n=16 | **Surveillance**  Progression rate: 9.5%  Mean time to relapse:  14.5 mo  PFS: 67/74 (90.5%)  OS: 100%  **Carboplatin**  Progression rate 12.5%  Mean time to relapse: 13.8 mo  PFS: 14/16 (87.5%)  OS: 100%  no statistical significance for outcomes | The authors declare that they have no conflict  of interest.  supported by the Slovak Research and Development Agency | LoE 4  RoB: not acceptable  Outcomes aren’t defined; No indication if or how measures were managed (regarding detection bias in outcome assessment);  No mention of confounding;  No confidence intervals given  *very small number of cases in Carboplatin-group* |
| Ondrusova  2017 | retrospective  cohort study  n=485  1992-2017  Slovak republic | To analyse a 25-year single center experience with risk-adapted therapeutic approaches – active surveillance versus adjuvant chemotherapy. | Non-seminoma clinical stage I  Low risk and high risk  Mean age  Surveillance: 30.1±7.9 y  Chemotherapy: 31.4±8.81 y | Active surveillance  (Low risk)  N=301 | Adjuvant chemotherapy  (High risk)  n=184  2xBEP | **Relapse rate**  Active surveillance  52/301 (17.3%)  median follow-up 7.2 mo  Adjuvant chemotherapy  2/184 (1.1%)  median follow-up 56.2 mo  Chemotherapy vs. Surveillance  HR 0.058 (0.014-0.239)  p<0.001  **OS**  Chemotherapy vs. Surveillance  HR 0.279 (0.033-2.324)  p=0.238 | The authors declare that they have no conflict of interest.  No information about funding | LoE 2b  RoB: acceptable  Groups differ significantly from each other (low risk and high risk*)*  *different follow-up periods for treatment groups and PFS, OS* |
| Ruf  2019 | retrospective cohort study  n=451 patients  1994-2014  Germany  Median follow-up  Surveillance: 40 mo  Carboplatin: 96 mo  Radiotherapy: 142 mo | To examine the health-related  events that occurred during follow-up, with a particular focus on patients who had undergone adjuvant  carboplatin treatment. | clinical stage I  testicular seminoma | Surveillance  n=127 | Carboplatin therapy  n=243  Radiotherapy  n=81 | **Treatment toleration**  Surveillance: 81.9%  Carboplatin: 71.2% (p=0.02)  Radiotherapy: 77.8% (p=0.5)  Subsequent malignant  neoplasms  Surveillance: 1/124  Carboplatin: 12/242  Radiotherapy: 7/81 | None of the authors declare any conflicts of interest  related to the present report.  no funding | LoE 4  RoB: not acceptable  17% of the originally identified patients were lost to follow-up; 30% did not respond to the questionnaire.  Different median follow-up intervals between the groups.  Implementation of the interventions is not described in detail.  The data set was generated from the responses of patients to the questionnaire and from third-party enquiries. |
| Shinoda  2018 | Retrospective cohort study  n=159 patients  2005-2008  Japan  Median follow-up:  30.3 mo (0.3-65.6 mo) | To evaluate the survival rate and risk factors of distant metastasis in stage I nonseminomatous germ cell tumor) cases without adjuvant treatments. | stage I non-seminomatous  germ cell tumor  Mean age  Surveillance: 33.6 ± 11.73 y  Adjuvant therapy: 31.8 ± 11.33 y | Surveillance  n=132 | Adjuvant treatment  n=27 (22 chemotherapy,  1 radiation,  3 RPLND,  1 chemotherapy+RPLND) | Relapses  Surveillance: 16/132  Adjuvant therapy: 1/27  2-y RFS  Surveillance: 90%  Adjuvant therapy: 96%  p=0.1548  Surveillance group (n=132)  Risk factors of distant metastasis  Age at orchiectomy, preoperative AFP levels and LDH level, pT classification and presence of yolk sac tumor components: no significance  embryonal carcinoma elements: 15 (19.2%)  no embryonal carcinoma elements: 1 (1.8%)  p=0.0068 | None declared. | LoE 2b  RoB: acceptable  Frequency and performance of follow-up investigations were not unified between the centers and described. |
| Tandstad  2010 | Prospective cohort study  n=232  July 1995-January 1998  Sweden  Norway  Median follow-up:  122 mo (91-138 mo) | To reduce the risk of relapse and thereby reducing the need of later salvage chemotherapy while maintaining a high cure rate. | clinical stage I non-seminomatous germ-cell testicular cancer  n=165 without vascular invasion in the testicular tumor  n=67 with vascular invasion in the testicular  Median age: 29.5 y (24.7-35.7 y) | Surveillance  n=129  n=5 with vascular invasion  n=124 without vascular invasion | Chemotherapy  n=103  n=62 with vascular invasion  n=40 without vascular invasion  n=1 uncertainty regarding vascular invasion | **Relapse**  Surveillance  with vascular invasion: 3/5  without: 16/124  Chemotherapy  with vascular invasion: 1/62  without: 4/40  unclear: 0/1  no statistical difference in relapse rate between 1x CBV vs. surveillance for patients without vascular invasion  2x CBV for patients with vascular invasion reduced the relapse rate with >90% in comparison to surveillance  **Toxicity for CBV**  90%–95% of all cases  (27% Grade 3/4) | no information about funding or conflict of interest | LoE 4  RoB: not acceptable  Patient recruitment not described in detail;  Recruitment into the study was terminated prematurely due to increasing toxicities;  Patient characteristics are not reported;  No mention of confounding  *pooled results of a RCT and a prospective cohort study* |
| Tandstad  2011 | prospective cohort study  n=1384  2000–2006  Norway, Sweden  Median follow-up:  Overall: 5.2 y  Surveillance: 5.0 y  Radiotherapy: 6.1 y  Carboplatin:  3.4 y | The aim was to standardize care for all patients with seminoma to further improve the good results expected for this disease. | patients with seminomatous testicular cancer  Mean age:  37 y | Surveillance  n=512 | Carboplatin  n=188  Radiotherapy  n=481 | **Relapse rate**  Surveillance  14.3% median time to relapse: 1.4 y  Carboplatin  3.9% median time to relapse: 1.8 y  Radiation  0.8% median time to relapse: 1.1 y  Carboplatin vs. Radiotherapy  HR 4.7 (95% CI 1.1-14.4) p=0.031  Surveillance vs. Carboplatin  HR 3.9 (95% CI 1.6-9.3)  p=0.02  **Relapse free interval**  Surveillance: 85.7%  1x Carboplatin: 96.1%  Radiation: 99.2%  **5-y OS**  Surveillance: 98.4%  1x Carboplatin: 99.2%  Radiation: 98.7%  **5-y CSS**  Surveillance: 99.8%  1x Carboplatin: 100%  Radiation: 100% | Swedish Cancer Society,  the Gunnar Nilsson Foundation for  Cancer Research, and the Nordic  Cancer Union.  The author(s) indicated no potential conflicts of interest. | LoE 2b  RoB: acceptable  No mention of confounding  *results shown only for clinical stage I* |
| Tandstad 2014 | prospective cohort study  n=1003  1995-2005  Sweden,  Norway  Median follow-up:  8.3 y | The aim of  this study is to report the incidence of intratubular germ cell neoplasia of unclassified type and  bilateral T testicular germ cell cancer and to evaluate the effect of adjuvant  chemotherapy on the risk of developing a metachronous  testicular germ cell cancer. | clinical stage I non-seminoma | Surveillance  n=494 | Adjuvant chemotherapy  n=494  cisplatin-based chemotherapy in combination with 1x CVB or BEP | **Incidence bilateral testicular germ cell cancer**  Surveillance: 13/494 (3.4%)  Chemotherapy: 11/494 (2.5%)  p=0.41 | The authors report no  conflicts of interest.  Founding:  National Cancer Fund of Sweden | LoE 4  RoB: not acceptable  No indication, what the relevant population characteristics were; Dropout rates are mentioned, but no follow-up information is provided; Performance of interventions is not described; No comment or analysis of potential impact on results. |
| Tandstad  2016 | prospective cohort study  n=1118  (n=897  SWENOTECA VII  2007-2010  and  n=221  SWENOTECA V)  Norway, Sweden  Median follow-up:  Surveillance:  5.4 y (4.5–6.3 y)  Carboplatin:  5.7 y (4.3-7.3y) | The protocol aimed to prospectively validate the proposed risk factors for relapse, stromal  invasion of the rete testis and tumor diameter >4 cm, and to evaluate the efficacy of one course of adjuvant carboplatin. | clinical stage I seminoma | Surveillance  (with no or one risk factor)  n=422  risk factors:  largest tumour diameter >4 cm and/or stromal invasion of rete testis | Carboplatin  (with two risk factors)  n=469 | **Median time to relapse**  Surveillance: 1.3 y (0.4-5.6 y)  Carboplatin: 1.7 y (0.2-6.5 y)  **5-y OS**  Surveillance: 99.2%  Carboplatin: 98.9%  **10-y OS**  Surveillance: 96.8%  Carboplatin: 98.5%  **5-y CSS**  Surveillance: 100%  Carboplatin: 100%  **10-y CSS**  Surveillance: 99.6%  Carboplatin: 100% | supported by Research Committee  at St Olavs Hospital, Trondheim, the Swedish Cancer Society,  the Swedish Association of Local Authorities and Regions, the Norwegian Regional Health Authorities, and the Norwegian Urological Cancer Group  The authors have declared no conflicts of interest. | LoE 4  RoB: not acceptable  no information about patient characteristics,  No mention of confounding  “Within the risk-adapted SWENOTECA VII protocol, 11.2% of the men had two risk factors, but in all 53% of the patients chose adjuvant carboplatin. Patients were informed of an estimated RR of 15% if no or one risk factor was present. This indicates that many well-informed patients may prefer adjuvant treatment even if the absolute risk of relapse is low. The proportion of patients choosing adjuvant carboplatin differed between hospitals, probable due to the physician bias regarding treatment options.” |
| Terbuch  2017 | retrospective cohort study  n=406  1994-2013  Austria  Median follow up:  8.6 y (21 days-21.6 y) | We assessed long-term cardiovascular complications  and identified risk factors for cardiovascular events in stage I seminoma patients. | clinical stage I seminoma  Mean age: 37.3 y (32.4-44.1 y) | Surveillance  n=312 | Adjuvant chemotherapy  n=37 patients  (single shot carboplatin)  Radiotherapy  n=57 | **Relapse**  Surveillance: 35/312 (11.2%)  Carboplatin: 3/37 (8.1%)  Radiotherapy: 1/57 (1.8%)  **Cardiovascular events**  Surveillance: 14/312  Carboplatin: 0/37 patients  Radiotherapy: 9/57 patients  Radiotherapy vs. Surveillance  Risk difference: 11.3% (95% CI 4.6-18%) p=0.001  Radiotherapy vs. Carboplatin  Risk difference: 16% (95% CI 6-25%) p=0.001 | None of the contributing authors have any  conflicts of interest  Founding:  Medical  University of Graz | LoE 2b  RoB: acceptable  “Our study has some limitations due to its retrospective nature of data collection and missing data. Furthermore, the shorter follow-up time of the patients treated with carboplatin has to be taken into account in the interpretations of our finding.” |
| Tyrrell  2018 | retrospective cohort study  n=501  2004-2016  United Kingdom | We present an analysis of the risk factors and relapse  rates for this cohort of patients, together with an analysis of how  recurrences were identified and managed. | clinical stage I seminoma | Surveillance  n=326 | Adjuvant treatment  n=175 | Relapses  Surveillance: 6.1%  Adjuvant treatment: 6.2%  Recurrence with tumors > 4 cm vs. ≥ 4 cm tumor size  Overall  HR 1.219 (95% CI 0.58-  2.55; p=0.598)  Surveillance group  HR 1.316 (95% CI 0.51-3.43; p=0.574)  Adjuvant treatment  < 4 cm tumor size: 107/345 (31%)  ≥ 4 cm tumor size: 68/156 (43.6%)  Recurrence and presence of rete testis invasion  Overall  HR 2.26 (95% CI 1.11-4.61;  p=0.025)  Only true stromal rete invasion  HR 3.00 (95% CI 1.47-6.14; p=0.003)  Only true stromal rete invasion  in the surveillance group  HR 3.35 (95% CI 1.38-8.11)  Markers a-fetoprotein, b-human chorionic gonadotropin, and lactate dehydrogenase  No statistically significant recurrence with positive tumor marker levels | The authors declare that they have no competing interests. | LoE 4  RoB: not acceptable  No indication of how groups were selected and what the relevant population characteristics were; Outcomes are not clearly defined. Performance of interventions and assessment of risk factors were not described; No mention of confounding.  *Median follow-up not reported* |
| Weiner  2017 | retrospective cohort study  n=6660  2004-2013  USA  (National Cancer Data Base)  Median follow-up:  45.0 mo  (25.1-69 mo) | To evaluate temporal trends in the management of early stage non-seminomatous germ cell tumor in a large, contemporary United States hospital-based cancer registry and to identify the patient and clinical factors associated with surveillance. | Non-seminomatous germ cell tumor clinical Stage IA  n=4080  non-seminomatous germ cell tumor clinical Stage IB  n=2580 | Surveillance  n=2873 clinical stage IA  n=1195 clinical stage IB | RPLND  n=676 clinical stage IA  n=503 clinical stage IB  Chemotherapy  n=531clinical stage IA  n=882 clinical stage IB | **Clinical stage IA**  5-y OS  Surveillance: 97.3% (CI 96.3-98.0)  RPLND: 99.1% (CI 97.6-99.7)  Chemotherapy: 98.0% (CI 96.2-99.0)  10-y OS  Surveillance: 94.2% (CI 91.2-96.2)  RPLND: 97.5% (CI 93.6-99.1)  Chemotherapy: 95.1% (CI 89.8-97.7)  p=0.064  **Clinical stage IB**  5-y OS  Surveillance: 96.5% (CI 94.8-97.7)  RPLND: 97.8% (CI 95.5-99.0  Chemotherapy: 96.0% (CI 94.1-97.3)  10-y OS  Surveillance: 95.8% (CI 93.4-97.4)  RPLND: 97.0% (CI 93.7-98.6): Chemotherapy  91.7% (CI 77.7-97.0)  p=0.411 | No specific funding or conflict of interestdisclosed. | LoE 2b  RoB: acceptable  Regime of chemotherapy is not described. Confounding is mentioned, but no comment or analysis of potential impact on results. |
| Yap  2017 | retrospective cohort study  n=3961  1988–2010  USA-California  Median follow-up:  96 mo | To assess the shifting population-level practice patterns across a 20-year time span in the management of  stage I non-seminomatous germ cell tumors. | stage I non-seminomatous germ cell tumors  Mean age: 28 y (23-35 y) | Surveillance  n=1903 | Chemotherapy  n=962  RPLND  n=1049 | **5-y OS**  Surveillance: 97%  Chemotherapy: 92%  RPLND: 98%  **5-y CSS**  Surveillance: 99%  Chemotherapy: 94%  RPLND: 99% | There are no conflicts of interest, either financial  or non-financial, for all of our contributing authors. | LoE 2b  RoB: acceptable  No indication if or how measures were managed (regarding detection bias in outcome assessment), descriptive comparison |

**Abbreviation**

BEP Bleomycin, Etoposide and Cisplatin

CBV Cisplatin, Vinblastine and Bleomycin

CI Confidence interval

CSS Cause-Specific Survival

CSM Cancer-Specific Mortality

DFS Disease-Free Survival

DSS Disease-Specific Survival

HR Hazard Ratio

LoE Level of Evidence

mo months

NSGCT Non-Seminomatous Germ Cell Testicular

OS Overall Survival

OCM Other-Cause Mortality

RFS Relapse-Free Survival

RFR Relapse Free Rate

RoB Risk of Bias

RPLND Retroperitoneal Lymph Node Dissection

SD Standard Deviation

y years
