# Supplementary material for: Testicular germ cell tumours’ clinical stage I: comparison of surveillance with adjuvant treatment strategies regarding recurrence rates and overall survival—a systematic review
Source: World J Urol. 2022 Sep 15;40(12):2889–900. doi: 10.1007/s00345-022-04145-6 (PMC9712330; doi:10.1007/s00345-022-04145-6)
Supplement: Supplementary file 2 — Supplementary file2 (DOCX 28 KB) [file 345_2022_4145_MOESM2_ESM.docx]

| Reference | **Design**  **Number of patients**  **Country**  Follow-up | Objective | Patients | Results | **Funding and conflict of interests** | **Level of evidence**  **Risk of bias**  *additional comments* |
| --- | --- | --- | --- | --- | --- | --- |
| Cummins, 2010 | Retrospective case series  n=164  1980-2004  United Kingdom  Median follow-up: 13.5 y (1-20 y) | To provide data to advise patients on treatment burden and risk of recurrence associated with surveillance. | stage I  seminoma patients | **Relapses**  22/164 (13%)  **Death**  Overall: 6/164  After relapse: 2/22  Unrelated**:** 4/142  **Disease-specific mortality**  1.3%  **Symptomatic at time of relapse**  4/22 (18%) | The authors have declared no conflicts of interest.  Funding: NHS Executive, Institute of Cancer Research, and Cancer Research UK grant number C46/A3970 to the ICR Section of Radiotherapy | LoE 4  RoB: not acceptable  patient characteristics only from relapsed patients described, it is not clearly stated whether all patients between 1980-2004 are included, descriptive analysis |
| Daugaard, 2014 | Retrospective case series  n=1226  1984-2007  Denmark  Median follow-up: 180 mo (1-346 mo) | To describe treatment results in a large cohort with stage I NSGCC treated in a surveillance program. | stage I NSGCC  Median age: 30 (15-79 y) | **Risk of relapse**  6-mo: 225/1226 (18.5%)  1-y: 304/1226 (24.9%)  2-y: 358/1226 (29.4%)  5-y: 376/1226 (30.6%)  **OS**  5-y: 97.6%  10-y: 96.2%  15-y: 94.5%  **DSS**:  5-y: 99.3%  10-y: 99.3%  15-y: 99.1% | The authors indicated no potential conflicts of interest  Financial support: Gedske Daugaard | LoE 4  RoB: acceptable  follow-up program changed little during the years |
| Kollmannsberger, 2015 | Retrospective case series  n=2483  1998-2010  Multinational  Median follow-up:  Seminoma  52 mo (1-322 mo)  Nonseminoma  62 mo (1-277 mo) | To evaluate the performance of active surveillance as a management strategy in broad populations and to inform the development of surveillance schedules by individual patient data regarding timing and type of relapse. | clinical stage I  n=1344 seminoma  n=1139 nonseminoma  Age:  Seminoma: 30 y (14-85 y)  Nonseminoma: 37 y (18-86 y) | **Relapses**  Seminoma: 173/1344 (13%)  Nonseminoma: 221/1139 (19%)  **Death**  Disease-related  Seminoma: 0  Nonseminoma: 3  Treatment-related complications  Seminoma: 1  Nonseminoma: 2  Cancer-unrelated causes  Seminoma: 16  Nonseminoma: 11  **Alive without disease**  Seminoma: 99%  Nonseminoma: 98%  **DSS**:  Overall  5-y: 99.7% (95% CI 99.24-99.93)  10-y: 99.7% (99.4%-99.9%)  Nonseminoma:  5-y: 99.4%  (95% CI 98.9%-99.93%)  10-y: not reported  Seminoma:  not reported | Honoraria: Tom Powles,  GlaxoSmithKline, Pfizer, Astellas Pharma  Research Funding: None | LoE 4  RoB: not acceptable  not clearly stated whether all patients between 1983-2010 are included  „Different schedules for active surveillance were employed at participating institutions […]“  *Slightly different numbers for 5-y DSS in abstract and Table 3* |
| Lago-Hernandez, 2015 | Retrospective case series  n=266  1997-2013  United States of America  Median follow-up:  42 mo (1–264 mo) | We carried out a retrospective survey to establish  a time-to-relapse model in the first 2 years of surveillance for clinical stage I nonseminoma germ-cell tumor patients according relapse-associated risk factors, with  particular emphasis on the first 6 mo. | patients with germ-cell tumors  n=131 seminoma  n=135 nonseminoma  Median age:  32 y (17-66 y) | **Relapses**  Overall: 70/266 (26%)  Seminoma: 20/131 (15%)  Nonseminoma: 50/135 (37%)  **Nonseminoma risk factor score of relapse**   - No lymphovascular invasion + no embryonal predominance: 19/76 (25%), HR 1.67 (0.89–3.15), p=0.108 - lymphovascular invasion or embryonal predominance: 17/41 (41%), HR 3.35 (1.75–6.40), p<0.001 - both: 14/18 (77%), HR 9.80 (4.91–19.56), p<0.001 | The authors have declared no conflicts of interest.  Financial support: Shawmut Design and  Construction’s Pan Mass Cycling Team | LoE 4  RoB: not acceptable  no standardized surveillance: The frequency of surveillance  […] was decided by the treating physicians and in essence followed NCCN guidelines at relevant times. |
| Nayan, 2017 | Retrospective case series  n=1239 patients  1980-2014  Canada  Median follow-up (those without relapse):  Seminoma: 88.4 mo (IQR 52.1–122.1)  Nonseminoma: 60.6 mo (IQR 34.1–99.1) | To determine conditional risk of relapse in clinical stage I testicular cancer. | n=775 seminoma  n=464 nonseminoma  Mean age  Seminoma: 37.00 y (SD 9.71)  Nonseminoma: 30.3 y (8.6) | **Risk of relapse within 5 years**   - seminoma with tumor size ≥3cm: 20.3% - seminoma with tumor size <3cm, respectively: 12.2% - Nonseminoma high-risk: 42.4% - Nonseminoma low-risk: 17.3% | The authors have declared no conflicts of interest.  Funding: None | LoE 4  RoB: not acceptable  only complete datasets were used for the analysis during the recruitment period, surveillance protocols have changed over time, table 1: seminoma patients n=775 does not match with the numbers under year of orchiectomy |

**Abbreviations**

CSS Cancer-Specific Survival

CI Confidence Interval

DSS Disease-Specific Survival

GCTC Germ Cell Testicular Cancer

HR Hazard Ratio

IQR Interquartile Range

LoE Level of Evidence

mo months

NCCN National Comprehensive Cancer Network

NSGCC Nonseminoma Germ Cell Cancer

OS Overall Survival

RFS Relapse-Free Survival

SD Standard Deviation

y years
